# Massive Gastrointestinal Bleeding Masquerading Coagulopathy in Acute Viral Hepatitis: A Case Report

**DOI:** 10.31729/jnma.5007

**Published:** 2020-06

**Authors:** Bhishma Pokhrel, Sunil Kumar Daha, Nikhil Shrestha, Pankaj Kumar Sah, Nimesh Khanal

**Affiliations:** 1 Patan Academy of Health Sciences, Lalitpur, Nepal; 2 Oxford University Clinical Research Unit-Nepal, Lalitpur, Nepal; 3 Kanchanjunga Hospital, Birtamode, Nepal

**Keywords:** *cholestasis*, *hepatitis A*, *meckel’s diverticulum*

## Abstract

Hepatitis A virus infection is typically an acute self-limiting illness associated with general nonspecific symptoms such as fever, malaise, anorexia, nausea, vomiting, abdominal pain or discomfort, and diarrhea. This may have atypical manifestation like prolonged cholestasis. Despite having varying typical and atypical manifestations such a case may present with life-threatening bleeding from a co-existing surgical cause such as perforation of Meckel's diverticulum.

## INTRODUCTION

Hepatitis A virus (HAV) infection is common, typically an acute self-limiting illness may have several atypical manifestations including hepatic and extrahepatic.^13^ We encountered a case of HAV infection which complicates with its atypical manifestation and become critical enough and shifted to the pediatric intensive care unit (PICU), where he developed uncontrolled massive lower gastrointestinal bleeding. Later the cause for bleeding was found to be due to Meckel’s diverticulum perforation diagnosed via exploratory laparotomy. With the lesson that a patient with viral hepatitis may have life-threatening bleeding from a co-existing surgical cause, we report this case to enhance awareness among medical practitioners.

## CASE REPORT

A 10-years old male child with uneventful birth, immunization, developmental, and past medical history from Kathmandu presented with fever for 5 days, yellowish discoloration of body, and vomiting for last three days in pediatric referral clinic (PRC). On the history of presenting illness, fever was intermittent, the maximum recorded temperature was 101.6degree Fahrenheit, responding to paracetamol, and not associated with chills or rigor. His parents noticed yellowish discoloration of eyes and face three days back and later progressed to all over the body. Yellowish discoloration of the body was associated with dark colored urine and pale stool. The child was completely anorexic and vomited with attempted feedings and was nauseated most of the time. There was a total of four episodes of non-projectile vomiting. The vomitus contained food particles of about 100 ml in each episode, neither bilious nor mixed with blood. There was no other significant systemic history related to this illness.

On examination, the general condition was fair and vital signs were within normal limits. General physical examination showed positive signs of pallor and icterus. Anthropometric parameters were within normal limits. The abdominal examination showed enlarged liver with a total span of 15cms in mid-clavicular line (it was palpable 3cm below the subcostal margin), the margin was round with smooth surface and tenderness was present. The child was admitted for persistent vomiting and had not taken oral feed except for sips of water and was treated with IV fluids, injection Vitamin K, syrup lactulose, and kept on liquid to semisolid diet.

Lab reports on the day of admission are shown (Table 1).

**Table 1:** Values of lab parameters.

Lab parameters	On the day of admission	On the fourth day of admission
Total leukocyte count and differentials	12500	
Neutrophils	76%	
Lymphocytes	21%	
Hematocrit (%)	37%	
Platelet (/microL)	277*10^3^	
Random blood sugar (mg/dl)	82	82
Urea (mmol/L)	13	28
Creatinine (mmol/L)	0.3	0.5
Sodium (mEq/L)	132	131
Potassium(mEq/L)	4.4	4.7
*SGPT (U/L)	6494	
nSGOT(U/L)	8706	
ALP(U/L)	300	
Albumin (mg/dl)	3.5	3.8
**PT/nnlNR	17.8/1.48	11.8/ 0.84
APTT	41	
Total serum bilirubin (mg/dl)	32	64.5
Direct serum bilirubin (mg/dl)	20	41.6

*SGPT: Serum glutamic pyruvic transaminase; nSGOT: Serum glutamic oxaloacetic transaminase; ALP: Alkaline Phosphatase **PT: Prothrombin time; nnlNR: International normalized ratio; APTT: Activated partial thromboplastin time.

**Figure 1:**
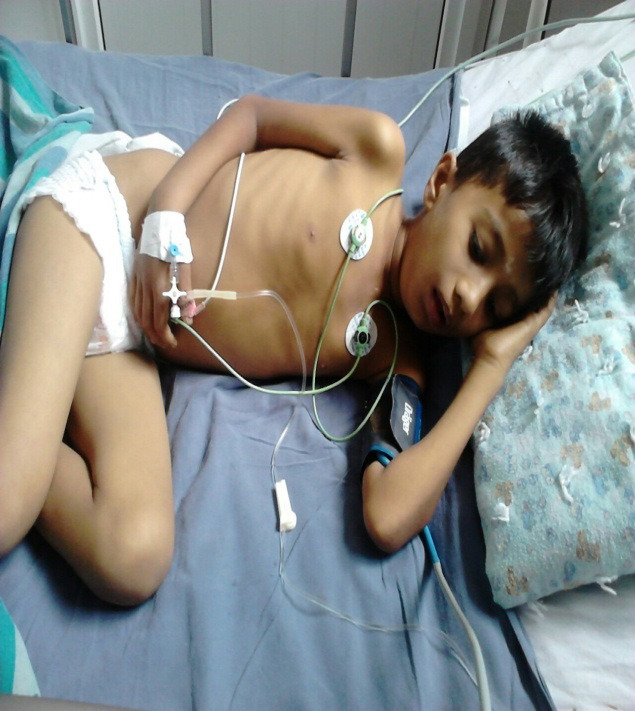
The child being monitored in the PICU.

During ward, stay child refused oral feeds, with persistent nausea and vomiting with attempted feeds. Moreover, yellowish discoloration of the body became highly pronounced and serology for viral etiology came positive for hepatitis A (i.e. HAV IgM antibody). At the time, lab report findings suggested conjugated hyperbilirubinemia (Table 2). Thus, the provisional diagnosis of acute viral hepatitis with cholestasis as an atypical manifestation was made. The child was then transferred to the Pediatric Intensive Care Unit (PICU) for close monitoring and to look for any signs of hepatic encephalopathy (Figure 1).

While ongoing treatment in PICU, his skin was lemon-yellow in color, nasogastric feeding was started but he would vomit often. On the second day of PICU admission, he appeared paler than before and often complained of vague abdominal pain and had deranged renal function tests. From the third day of PICU admission, the child started passing fresh blood in the stool. Repeated blood tests showed a fall of hematocrit and decreased pattern of serum bilirubin and liver enzymes along with deranged renal function tests (Table 2).

**Table 2:** Lab parameters in ward and PICU stay

Date in AD (8/2018)	CBC (×10*3/ul)	Hb	Platelets (×10* 3/ul)	TSB (mg/ dl)	DSB (mg/ dl)	SGPT (U/L)	PT (sec)	AP TT (sec)	INR	Urea (m mol/L)	Creatinine (m mol/L)	Sodium (mEq/L)	Potassium (mEq/L)
17th				57	30	1513	12	21	0.1	21	1.2	137	4.3
19th	31.3	4.6	543	23.4	18.2	605	11	31	0.9	30	2.3		
20th										35	2.6		
21st	18.1	7.6	423	13.8		66				37	2.6	137	3.3
22nd	19.2	6.7	278	11.3	5.5	50	12	26	1.0	34	2.5	142	3.3
23rd			13.5										
24th	19.4	9.4	111	12.6		67				50	2.0		
26th	17.9	9.8	220	9.8		64				40	1.4	3.3

Subsequently, massive and frequent per rectal bleeding occurred, and the examination revealed active fresh bleeding per rectum. Whole blood, packed cells, platelets rich plasma, and fresh frozen plasma were provided. Despite vigorous management, there was life-threatening persistent bleeding which demanded inotropes and multiple blouses of crystalloids. Surgical, nephrology, and gastroenterology experts were consulted for deranged liver function test (LFT), renal function test (RFT), and severe episodes of bleeding.

**Figure 2:**
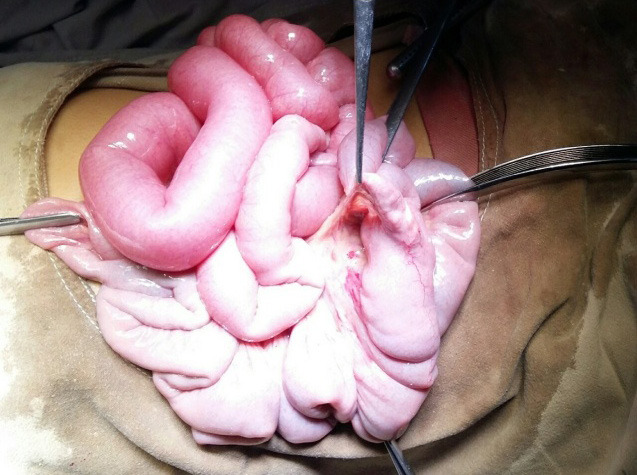
Small bowel diverticulum with perforation approximately 60cm from the ileocolic junction at antimesenteric border suggestive of Meckel’s diverticulum.

Exploratory laparotomy was performed as per surgical advice and the bleeding source was identified to be perforated Meckel’s diverticulum. The surgical findings identified small bowel diverticulum approximately 60cm proximal to the ileocolic junction at the antimesenteric border suggestive of Meckel’s diverticulum (Figure 2).

**Figure 3:**
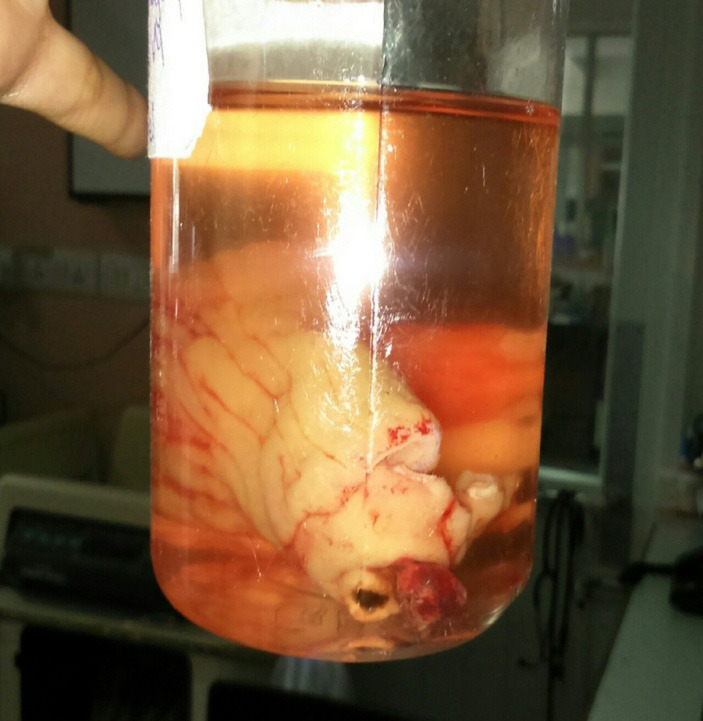
Specimen sent for histopathology.

The cut section showed an ulcer adjacent to the lumen of the diverticulum. Thus around 10 cm of ileum along with the diverticulum was removed and end to end anastomosis was done. The resected sample was sent for a histopathology examination (Figure 3).

Post-operatively the child was transferred back to PICU and kept on a ventilator for a few hours.

The gross findings of histopathological specimen conclude specimen of intestine measuring 9x2 cm with a diverticulum measuring 2.5x1cm and microscopic findings of the section from Meckel’s diverticulum showed gastric mucosa, foveola and surface epithelium lined by tall columnar cells with underlying lamina propria containing gastric glands mainly consisting of parietal cells and inflammatory cells; lymphocytes, plasma cells, and eosinophils. Section from intestinal mucosa was unremarkable. Conservative management was continued. On 3rd postoperative day, the child was transferred to the children’s ward and intravenous antibiotics continued, alternate day dressing was recommended. The stitch from the laparotomy incision site was removed on the seventh postoperative day and the child was discharged.

## DISCUSSION

Hepatitis A virus (HAV) is a member of the genus hepatovirus in the family Picornaviridae.^1^ Globally, an estimated 1.4 million cases occur each year.^1^It is typically an acute self-limited illness associated with general non-specific symptoms such as fever, malaise, anorexia, nausea, vomiting, abdominal pain or discomfort, and diarrhea.^2,4,5^ In this case report, a 10 years old child presented in PRC with fever, anorexia, yellowish discoloration of body and vomiting that developed within five days which were typical presentations of acute viral hepatitis A. HAV infection may also present with several atypical manifestations including fulminant hepatitis, relapsing hepatitis, prolonged cholestasis, and extrahepatic manifestations.^2,3^ In this case, we encountered an atypical extraintestinal manifestation of acute viral hepatitis presenting as life-threatening lower gastrointestinal bleeding due to perforated gastric mucosa present in Meckel’s diverticulum masquerading as coagulopathy. Meckel’s diverticulum (MD) is the most common congenital anomaly of the gastrointestinal tract with an estimated prevalence of 2% in the general population.^6^ Meckel’s diverticulum is caused by the failure of closure of the vitelline duct in the 5th week of fetal growth.^5,6^ Meckel’s diverticulum cases are generally asymptomatic, and complications and symptoms are more common in patients with ectopic tissue.^4,5,7^ The male to female ratio in Meckel’s diverticulum (MD) is one with male dominance in symptomatic cases.^4^ The presentation of MD was rectal bleeding which was found to be in the majority of cases of MD. A 20-year review study by St. Vil, et al. showed among 164 patients, 45 presented with bleeding symptoms and 60% of which were less than five years old.^8^ In a review of 158 infants less than one-year-old with lower gastrointestinal tract bleeding, only six presented with bleeding MD.^9^ Brookes, et al. reported nine months as the youngest among 43 children with MD.^10^ Rutherford, et al. showed that 43 out of 80 patients with symptomatic presentation had a bleeding MD but no age range was available. Poley, et al. study concluded that MD is rare among infants, and their patient was only the second 4-month-old infant.^9^ Studies show almost 50% of bleeding from an MD, occurs before the child is two years of age.^11^ From the above discussion, we can conclude that MD commonly presents in the pediatric age group and the presentations of MD vary from bleeding, abdominal pain, and intestinal obstruction.^6,12,13^ It is really difficult to diagnose MD in asymptomatic children.^6,12^ Focusing on this particular case where the HAV infection with prolonged cholestasis as atypical manifestation was the presumptive diagnosis. MD perforation was masquerading in this case and was an unexpected event in the course of treatment of this particular child. There was a dearth of literature and case reports where such a condition occurs. We did not find a single case report where there is acute viral hepatitis and perforated MD that occurred in the same setting. There is no prior evidence for the concurrence of these two conditions, thus the lower gastrointestinal tract bleeding as atypical manifestation with viral hepatitis is difficult for a clinician to diagnose or think about in such cases. This case gives us an educational value that we do have to think about possible co-existing surgical causes as well and investigate accordingly in the future. In this particular case, there was a delay in diagnosis and no investigations were sent pre-operatively to look for MD or other surgical causes. A patient with viral hepatitis may have life-threatening bleeding from a co-existing surgical cause. It is difficult to diagnose Meckel’s Diverticulum in asymptomatic child and even more difficult to diagnose when presenting with bleeding in case of acute viral hepatitis. Early diagnosis of the co-existing surgical cause in acute viral hepatitis can be managed safely but delay or missed diagnosis of such a case can be life-threatening.

**Consent: JNMA** Case Report Consent Form was signed by the patient and the original is attached to the patients’ chart.

## Conflict of Interest:

**None.**
